# Challenging Open Extraction of Intraarticular Intracapsular Broken Patellar Cerclage Wire Adjacent to the Medial Femoral Condyle Following Unsuccessful Arthroscopic Removal

**DOI:** 10.7759/cureus.58455

**Published:** 2024-04-17

**Authors:** Prashant Bhavani, Mainak Roy, Deepanjan Das, Samir Dwidmuthe, Sumit Raghute

**Affiliations:** 1 Orthopaedics, All India Institute of Medical Sciences, Nagpur, Nagpur, IND

**Keywords:** open surgical intervention, intraarticular complications, arthroscopic removal, cerclage wire migration, patellar fracture

## Abstract

Cerclage wiring and tension band wiring are commonly utilized in orthopedic surgeries for patellar fractures, but wire breakage is a recognized complication. This report presents a rare case where a broken cerclage wire exhibited intraarticular intracapsular migration, prompting open removal adjacent to the medial femoral condyle after unsuccessful attempts at arthroscopic extraction. A 50-year-old male with a history of patellar fracture fixation using cerclage and tension band wiring, presented with persistent knee pain and restricted motion. Radiographs revealed a united patellar fracture with a broken cerclage wire, and 3D CT pinpointed the wire fragment in the posterior knee compartment. Arthroscopic removal attempts through standard portals were ineffective, leading to a subsequent open removal via a Burk and Schaffer approach. Intraoperative fluoroscopy guided the thorough dissection, exposing the broken wire deep within the joint capsule, proximal to the intercondylar notch and adjacent to the medial femoral condyle. Meticulous extraction mitigated potential risks of cartilage and neurovascular damage. Follow-up imaging confirmed successful wire removal, and the patient experienced satisfactory functional recovery without significant complications. This case highlights the rare occurrence of intraarticular intracapsular migration of a broken cerclage wire and underscores the importance of timely removal to mitigate risks of cartilage and neurovascular damage. While arthroscopic removal is generally successful, cases of failure may necessitate open extraction, particularly when the wire is located posteriorly. The described approach, assisted by intraoperative fluoroscopy, proved effective in safely removing the broken wire and ensuring optimal patient outcomes.

## Introduction

Patellar fractures often require surgical fixation, commonly utilizing cerclage wiring and tension band wiring. However, complications such as wire breakage can occur. Various complications can also occur due to the migration of the broken wire. Broken wires may migrate towards the surrounding soft tissue, causing damage to the important neurovascular structures [1]. Migration of the wire to other major organs like the heart can cause potentially fatal complications [2]. To our knowledge, very few instances of intraarticular intracapsular migration of the wire have been documented [3]. This case report details the open surgical removal of a broken patellar cerclage wire directly adjacent to the medial femoral condyle, following an unsuccessful attempt at arthroscopic extraction, addressing the challenges and outcomes associated with this uncommon complication.

## Case presentation

**Figure 1:**
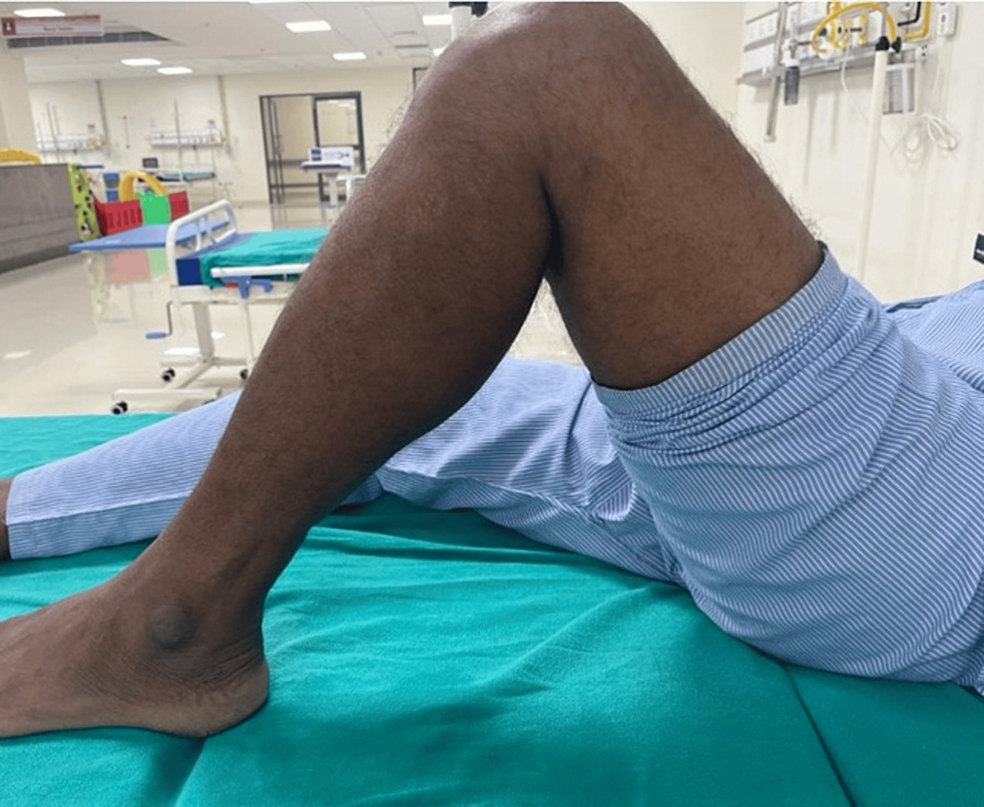
Preoperative clinical image showing restricted range of motion of the left knee.

A 50-year-old male presented to us with persistent pain in the left knee with a restricted range of motion. The patient had a history of patellar fracture fixation surgery using cerclage and tension band wiring 10 years ago. The patient was apparently normal till two weeks ago, after which he started developing pain in the left knee. The pain was deep-seated, non-radiating, aggravated on movement, and relieved at rest and with medications. There was no recent history of any fall or trauma to the left knee. On examination, there was tenderness over the left patella and the posterior aspect of the left knee. A mild increase in local temperature was also noted, along with a painfully restricted knee range of motion (Figure 1).

**Figure 2:**
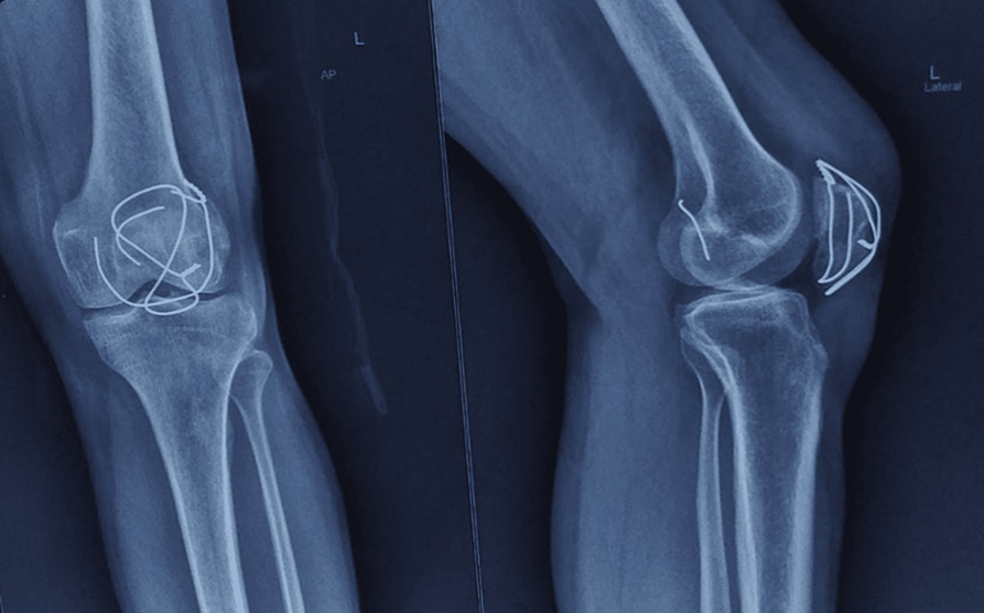
Preoperative radiograph showing a broken superomedial segment of the wire, migrated posteriorly.

There were no neurovascular deficits. The midline scar of the previous operation was healthy. Radiographs revealed a united patellar fracture with a broken cerclage wire. The superomedial portion of the wire had migrated to the posterior compartment of the knee joint (Figure 2).

**Figure 3:**
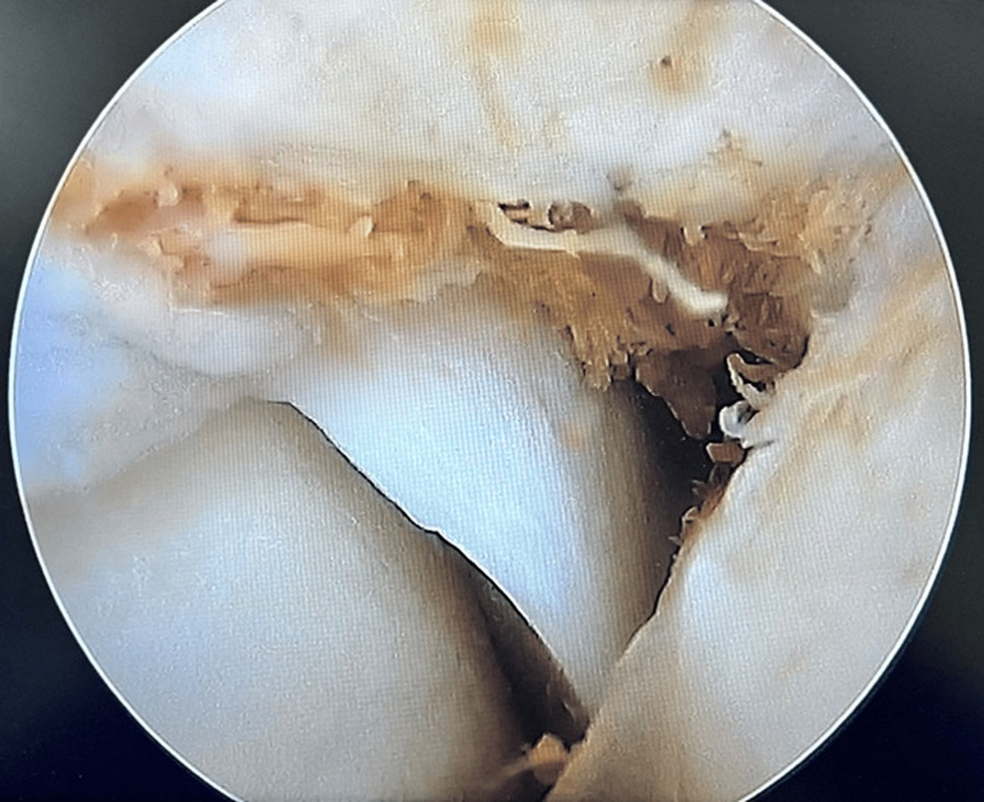
Diagnostic arthroscopy only revealed chondral changes over the medial patellar facet. There was a failure to visualize the wire.

The wire fragment within the posterior knee compartment was identified by 3D CT. The patient was then posted for the arthroscopic removal of the broken wire segment. Standard anteromedial and anterolateral portals were made. Diagnostic arthroscopy revealed high-grade chondral changes. A posteromedial portal was also made in an attempt to visualize the wire (Figure 3).

**Figure 4:**
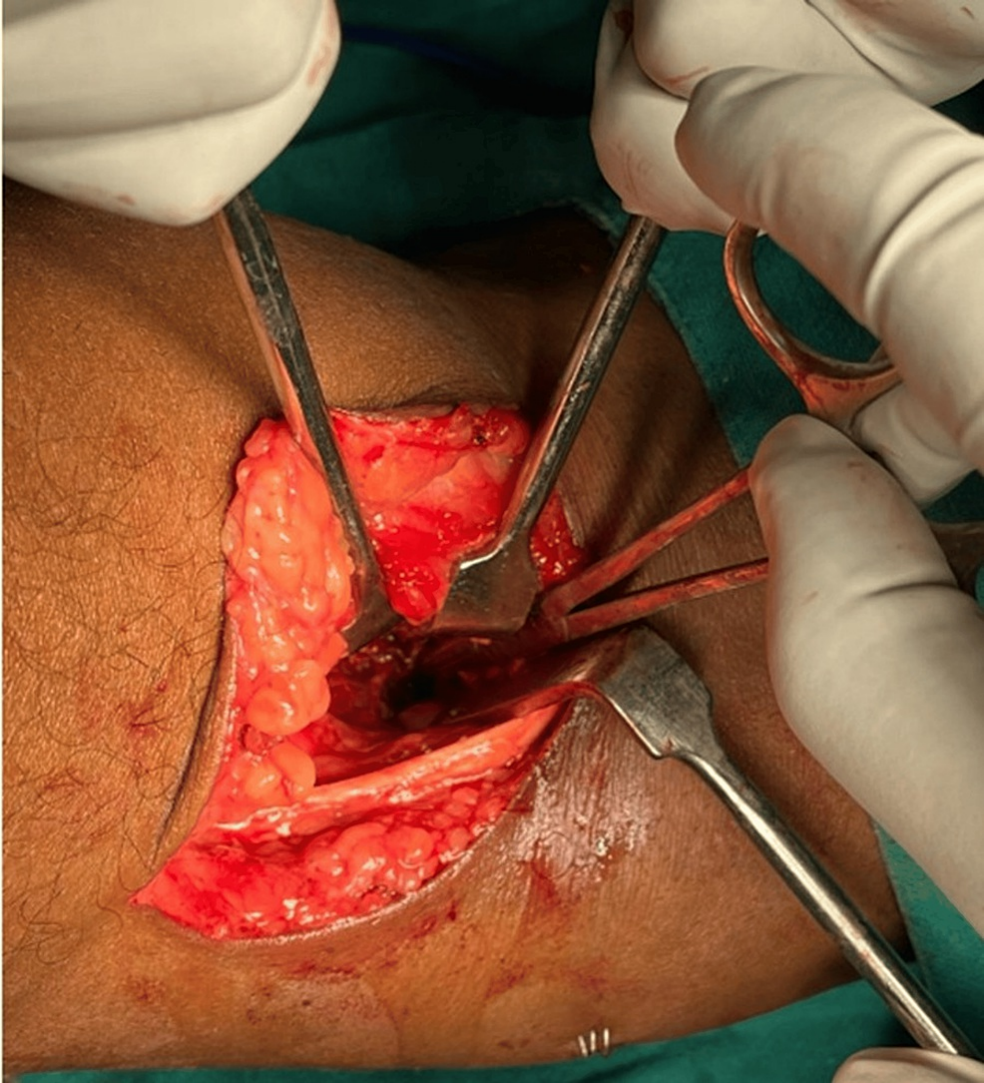
Approach and further dissection.

The attempt failed, despite using intraoperative fluoroscopy to guide us to the wire’s location. The soft tissues between the anterior cruciate ligament and the posterior cruciate ligament were cleared and it was tried the explore the area behind the posterior cruciate ligament (PCL). However, there was extensive fibrosis in the knee, which hindered our visualization of the broken wire. The intact segment of the wire was removed through a midline anterior incision over the previous scar. The patient was counseled thereby and again posted for open removal two days later. Open removal was performed through a Burk and Schaffer approach. With the patient in a prone position, a gentle, curved skin incision was placed over the popliteal fossa. The dissection was between the medial gastrocnemius and semimembranosus (Figure 4).

**Figure 5:**
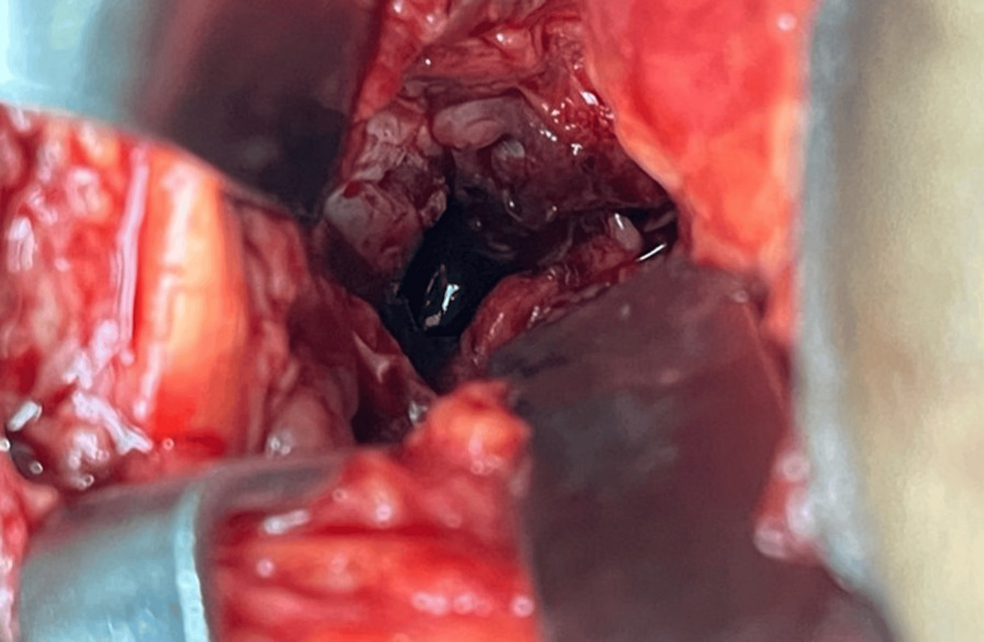
Burk and Schaffer approach taken; the wire's tip is visible.

The posterior capsule of the knee joint was identified and incised, revealing the broken wire deeply embedded inside the joint capsule, proximal to the intercondylar notch and adjacent to the medial femoral condyle, lying on the bone (Figure 5).

**Figure 6:**
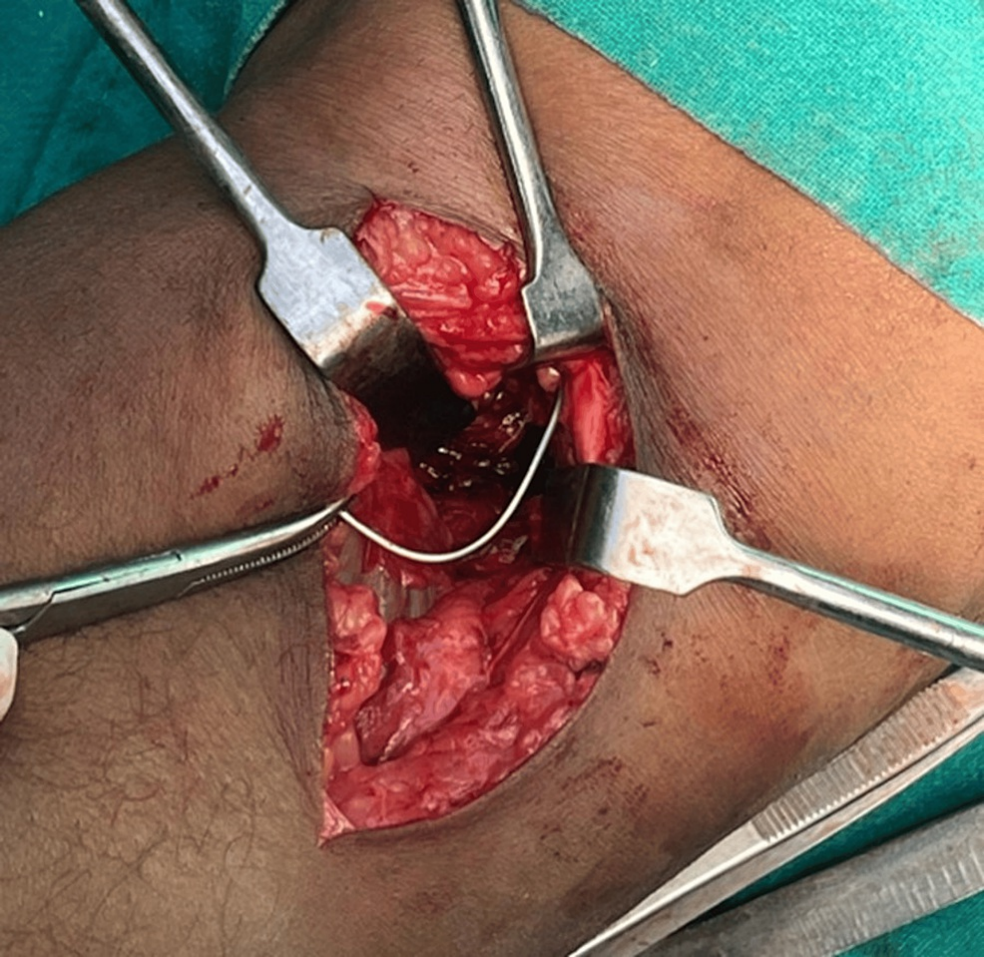
Broken wire segment successfully extracted.

Careful dissection was employed to expose and extract the fractured wire segment, minimizing the risk of additional damage to articular surfaces and surrounding soft tissues (Figure 6).

**Figure 7:**
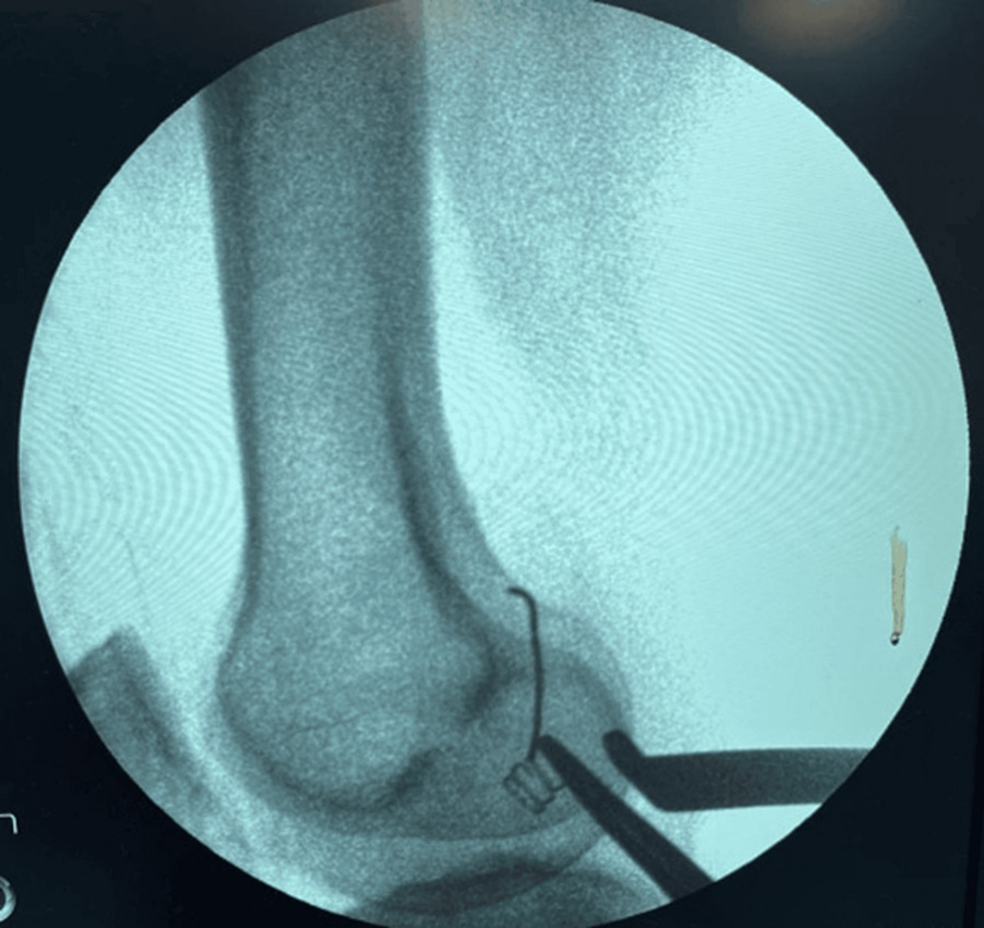
Intraoperative fluoroscopic guidance used for locating the broken wire.

Intraoperative fluoroscopy was used as a guide to the location of the wire (Figure 7).

**Figure 8:**
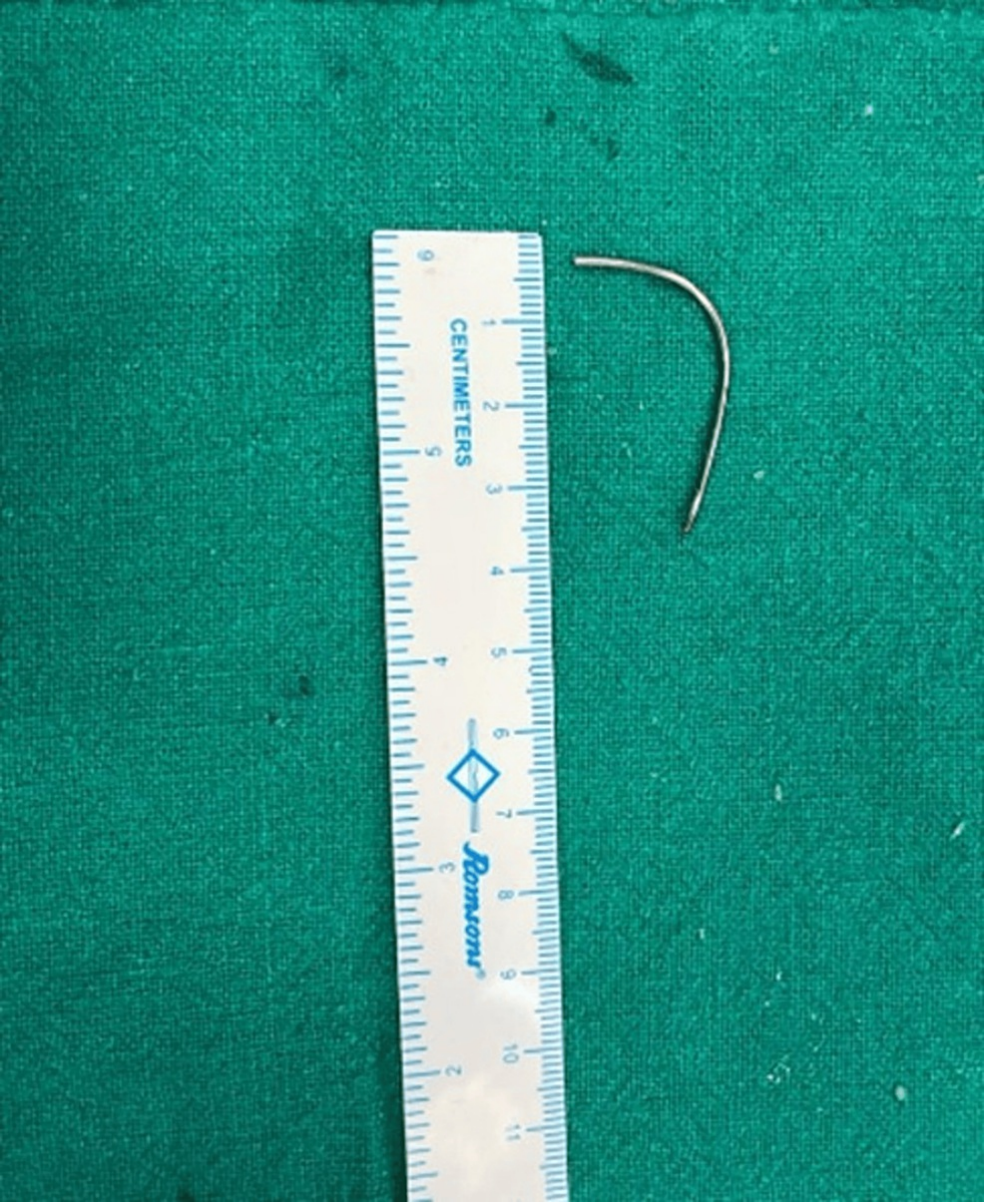
Extracted broken wire fragment.

A broken stainless steel wire segment of length around 4 cm was extracted (Figure 8).

**Figure 9:**
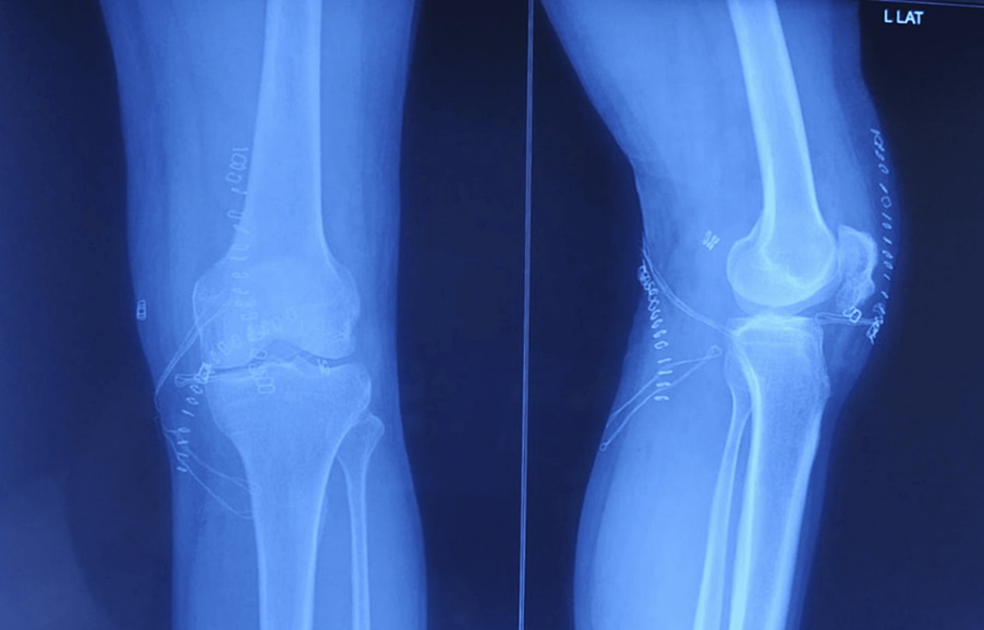
Postoperative radiograph showing successful wire extraction.

Postoperative radiographs confirmed successful wire removal (Figure 9).

**Figure 10:**
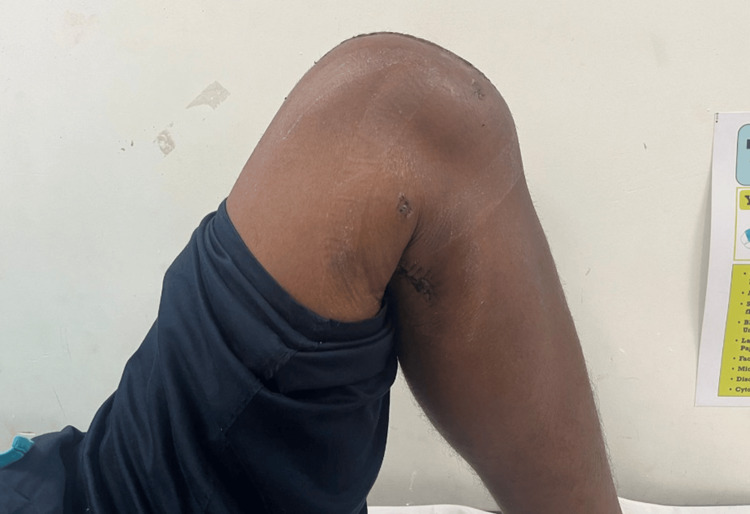
Postoperative clinical image showing a full range of motion.

The patient experienced satisfactory functional recovery and resumed normal activities without significant complications. He was pain-free and the range of motion had normalized (Figure 10).

## Discussion

Repetitive loading forces and the strain of both quadriceps and patellar tendon, even after the fracture unites, predispose to wire breakage [1]. There are very few reported cases of patellar wire migration in the literature. Only a few cases of intra-articular migration of a patella wire have been documented [3,4]. Our case is extremely rare in the sense that the wire had migrated to the posterior compartment of the knee joint, and that it was not possible to be located on arthroscopy, even with the assistance of intraoperative fluoroscopy. The major factor that played a role in the failure of the arthroscopic attempt was the extensive fibrosis present in the knee. The widespread fibrosis was possibly a result of the previous traumatic insult to the knee. Despite a posteromedial portal and even after exploring the area behind the PCL, it was not possible to visualize the broken wire segment. Thus, we were prompted to change our surgical plan and attempt an open removal.

We chose a simplified method to minimize the risk of damaging neurovascular structures, which is a concern in other procedures. By dissecting along the medial border of the medial head of the gastrocnemius and gently retracting laterally, we safely exposed the posterior capsule, protecting the neurovascular structures. This approach prioritizes safety and reduces the likelihood of unintended complications associated with the procedure [5]. Hence, when surgeons utilize K-wires or cerclage wires, they need to be mindful of potential complications arising from both local and distant migration of fractured wires. These complications can manifest as asymptomatic conditions, migration into joints causing cartilage damage, impingement on neurovascular structures resulting in pain, or even injuries to neurovascular structures like the popliteal artery, leading to severe hemorrhage. There is also a risk of broken wire fragments traveling through the venous system, reaching as far as the heart and causing tamponade. To prevent unnecessary complications associated with hardware failure, it is imperative for the surgeon to remove sharp hardware like K-wires and cerclage wires once the fracture of the patella has successfully united. In cases where the broken wire segment has migrated into the posterior compartment of the knee, arthroscopic removal can be attempted. If arthroscopy fails to visualize and extract the broken segment, open removal via the approach described above serves as an efficient and safe option for removal.

## Conclusions

This case report highlights the exceptional rarity of intraarticular intracapsular migration of a broken patellar cerclage wire, posing diagnostic and management challenges. Despite an initial unsuccessful attempt at arthroscopic removal, the subsequent open surgical intervention, utilizing a simplified and safe approach, successfully addressed the complication. The presented case emphasizes the need for surgeons to be vigilant regarding potential complications arising from wire migration, emphasizing the importance of timely hardware removal to prevent adverse outcomes. We attempt to enlighten orthopedic surgeons that extensive intraarticular fibrosis can hinder the visualization of the broken wire fragment and in case they are faced with a similar situation, it is always a wise idea to preoperatively counsel the patients for an open removal. The documented surgical approach provides valuable insights for clinicians facing similar complexities, contributing to the understanding and management of this uncommon complication.
